# Draft Genome Sequence of *Aeromonas salmonicida* subsp. *achromogenes* AS03, an Atypical Strain Isolated from Crucian Carp (*Carassius carassius*) in the Republic of Korea

**DOI:** 10.1128/genomeA.00791-13

**Published:** 2013-10-03

**Authors:** Jee Eun Han, Ji Hyung Kim, Sang Phil Shin, Jin Woo Jun, Ji Young Chai, Se Chang Park

**Affiliations:** Laboratory of Aquatic Biomedicine, College of Veterinary Medicine and Research Institute for Veterinary Science, Seoul National University, Seoul, Republic of Koreaa; Korea Institute of Ocean Science & Technology, Ansan, Republic of Koreab; Department of Rheumatology, Bundang Jesaeng Hospital, Seongnam, Republic of Koreac

## Abstract

We present the draft genome sequence of *Aeromonas salmonicida* subsp. *achromogenes* strain AS03, an atypical *A. salmonicida* strain that causes erythrodermatitis in crucian carp (*Carassius carassius*). This is the first genome sequence report of *A. salmonicida* subsp. *achromogenes*, one of the four subspecies of atypical *A. salmonicida*.

## GENOME ANNOUNCEMENT

*Aeromonas salmonicida* is a fish pathogen that causes furunculosis and other related diseases, and it is responsible for significant economic loss in the aquaculture industry (1–3). Five subspecies within *A. salmonicida* have been described: *A. salmonicida* subsp. *salmonicida*, *A. salmonicida* subsp. *achromogenes*, *A. salmonicida* subsp. *masoucida*, *A. salmonicida* subsp. *smithia*, and *A. salmonicida* subsp. *pectinolytica* (3, 4). *A. salmonicida* subsp. *salmonicida* is referred to as typical, whereas the other *A. salmonicida* strains are referred to as atypical (5).

The *A. salmonicida* subsp. *achromogenes* isolate AS03, which was collected from an ulcer in a crucian carp (*Carassius carassius*), was previously confirmed as *A. salmonicida* subsp. *achromogenes* (6), and genomic DNA was isolated using the DNeasy blood and tissue kit (Qiagen, Valencia, CA). Draft genome sequencing was performed using the Roche/454 pyrosequencing method on the Genome Sequencer FLX system with Titanium chemistry at Macrogen in Korea (21× coverage). Putative open reading frames (ORFs) were predicted using the Web-based NCBI Glimmer 3 tool (7), and translated ORFs were then compared to known protein sequences using BLAST (8). Gene ontology (GO) databases were additionally used to functionally classify the ORFs.

The 160,381 reads generated, with a length of 101,019,529 bp, were then assembled using *de novo* software (version 2.6). This assembly generated 69 large (>500 bp) contigs, with a length of 4,958,383 bp. The N_50_ contig length is 124,543 bp, and the largest contig assembled was 247,214 bp. The average contig size is 71,860 bp. The reads were assembled into 9 scaffolds with a length of 5,018,830 bp. The N_50_ scaffold was 2,773,112 bp, and the average length of the scaffolds is 557,647 bp.

The GO results revealed that 36%, 34%, and 9% of sequences included genes related to biological processes, molecular functions, and cellular components, respectively. In the GO category of biological processes, “metabolic processes” was the predominant subcategory, representing 33% of the genes. In the cellular component category, 48% of the genes were annotated as unknown, but 23% and 17% of the genes were associated with cell parts and membrane, respectively. Based on their molecular function, 48% of the genes were identified as being associated with catalytic activity.

Until now, some genome sequence data for typical *A. salmonicida* subsp. *salmonicida* strains, such as 01-B526 (9) and A449 (10), have been reported, but there have been no data for atypical *A. salmonicida* strains that cause atypical furunculosis and other related diseases in fish.

### Nucleotide sequence accession number.

The nucleotide sequence for the draft genome was deposited in GenBank under the accession no. AMQG00000000.
